# Frailty in Cardiac Surgery—Assessment Tools, Impact on Outcomes, and Optimisation Strategies: A Narrative Review

**DOI:** 10.3390/jcdd12040127

**Published:** 2025-03-31

**Authors:** Ashwini Chandiramani, Jason M. Ali

**Affiliations:** 1 Department of Cardiology, Royal Papworth Hospital, Cambridge, CB2 0AY, UK; ashwini.chandiramani@nhs.net; 2 Department of Cardiac Surgery, Royal Papworth Hospital, Cambridge, CB2 0AY, UK

**Keywords:** frailty, cardiac surgery, perioperative interventions, optimisation

## Abstract

Background: Advancements in surgical care have made it possible to offer cardiac surgery to an older and frailer patient cohort. Frailty has been recognised as a prognostic indicator that impacts post-operative recovery and patient outcomes. The aim of this study is to identify frailty assessment tools, evaluate the impact of frailty on post-operative outcomes, and explore strategies to optimise care for frail patients undergoing cardiac surgery. Methods: A comprehensive literature search was performed across PubMed, MEDLINE, and SCOPUS to identify articles reporting post-operative outcomes related to frail patients undergoing cardiac surgery. Results: Measurement tools such as gait speed, the Clinical Frailty Scale, Fried frailty phenotype, deficit accumulation frailty index and the Short Physical Performance Battery can be used to assess frailty. Frailty has been reported to increase the risk of post-operative morbidity and mortality. Multiple studies have also reported the association between frailty and an increased length of intensive care unit and hospital stays, as well as an increased risk of post-operative delirium. It is important to perform a comprehensive frailty assessment and implement perioperative optimisation strategies to improve outcomes in this patient population. Pre-operative strategies that can be considered include adequate nutritional support, cardiac prehabilitation, and assessing patients using a multidisciplinary team approach with geriatric involvement. Post-operatively, interventions such as early recognition and treatment of post-operative delirium, nutrition optimisation, early planning for cardiac rehabilitation, and occupational therapy can support patients’ recovery and reintegration into daily activities. Conclusions: The early identification of frail patients during the perioperative period is essential for risk stratification and tailored management strategies to minimise the impact of frailty on outcomes following cardiac surgery.

## 1. Introduction

The growing number of ageing patients with increasing comorbidities together with advancements in surgical care have made it possible to offer cardiac surgery to an older and frailer patient cohort [1]. Progressive ageing is associated with frailty, a state that can be characterised by an increased vulnerability to stress due to reduced physiological reserves which can result in a reduced ability to maintain or recover homeostasis following a destabilising event, such as cardiac surgery [2,3,4]. At a molecular level, ageing stems from the gradual build-up of cellular damage over time, which results in a progressive decline in physical and cognitive abilities. As a result of the processes associated with ageing, patients are more susceptible to frailty, sarcopenia, systemic impairments, reduced physical capacity, and functional independence [5]. Whilst physical strength and endurance are important aspects of frailty, it is a multidimensional dynamic syndrome that is also impacted by other factors of daily living, including a patient’s emotional state and cognitive function [3].

Frailty has been recognised as a prognostic indicator that impacts recovery trajectories and long-term outcomes in patients undergoing cardiac surgery. A recent meta-analysis involving 16,679 patients reported a two- to five-fold increase in post-operative complications, mortality risk, and hospital readmissions in frail patients who underwent cardiac surgery [2]. An updated meta-analysis assessing the prevalence of frailty and pre-frailty in 626,863 patients undergoing cardiac surgery estimated a pooled incidence of 28% frail and pre-frail patients in this patient cohort [1]. Despite surgical innovations and advancements in surgical technology, the decision on whether to proceed with invasive interventions in this patient population remains a challenge due to an increased risk of perioperative-related complications [6]. Less invasive procedures, such as transcatheter aortic valve implantation (TAVI) have developed as an alternative to surgical aortic valve replacement (sAVR) for high-risk patients, who are often older with multiple comorbidities [6]. Given the increasing prevalence of frailty among patients undergoing procedures such as coronary artery bypass grafting (CABG) and valve replacements, it is important to develop a better understanding of the perioperative care of frail patients undergoing cardiac surgery [3]. The aim of this study is to identify frailty assessment tools, evaluate the impact of frailty on post-operative outcomes, and explore strategies to optimise care for frail patients undergoing cardiac surgery.

## 2. Methods

We conducted a comprehensive literature review across various databases on PubMed, MEDLINE, and SCOPUS to identify English-language articles in the medical literature documenting post-operative outcomes related to frail patients undergoing cardiac surgery. The search timeframe covered the interval from inception up to December 2024. The specific search terms used were (‘Frailty’ OR ‘Frail’) AND (‘Cardiac Surgery’ OR ‘Heart Surgery’). Additional studies were identified by analysing the reference lists of pertinent articles.

## 3. Risk Stratification in Cardiac Surgery

The European System for Cardiac Operative Risk Evaluation (EuroSCORE) II (2010) and the STS score are the two widely used scoring systems for assessing perioperative mortality risk in cardiac surgery. Whilst EuroSCORE II and STS score focus on medical diagnoses and co-morbidities, a factor that is not accounted for is the patient’s biological status and presence of frailty. This in part reflects a lack of consensus on how to measure frailty and therefore data are not available for analysis and inclusion in these risk-scoring systems. EuroSCORE II was developed on a patient population with a mean age of 64.9 years [6,7,8]. Based on the Society for CardioThoracic Surgery of Great Britain and Ireland’s (SCTS) activity and outcomes report, the percentage activity for octogenarians in a cardiac surgery unit was estimated to rise from 7.2% (in 2002–2016) to almost 12% over the next 15 years [9]. EuroSCORE II currently incorporates ‘neurological or muscular dysfunction severely affecting mobility’; however, this is usually interpreted to include mechanical reasons for poor mobility rather than ‘frailty’ per se. Whilst some studies have reported that EuroSCORE II should be used with caution in patients >70 years of age due to an overestimation of surgical risk, a study evaluating the additional assessment of frailty using the comprehensive assessment of frailty test compared to the EuroSCORE and STS score described a low-to-moderate significant correlation between the frailty score and STS and EuroSCORE. They also reported a significant correlation between the frailty score and observed 30-day mortality [7,8,10]. Integrating a frailty score with traditional scoring systems has the potential to complement them, offering a promising avenue to create a refined and comprehensive risk stratification tool to improve their accuracy in older patients [11]. At present, the EuroSCORE III is being developed to account for the evolution of surgical techniques to reflect the current patient demographics undergoing cardiac surgery and hopes to include frailty; however, this is dependent on hospitals contributing data to record frailty routinely in their patients.

## 4. Measurement Tools to Assess Frailty

Frailty was traditionally assessed subjectively using an ‘end-of-the-bed test’, in which a patient’s frailty was evaluated using clinical judgement based on their visual appearance. While this approach can be useful, frailty may not always appear visually evident, and ‘apparent frailty’ can fluctuate based on the environment (hospital, community, clinic), time of day, nutritional status, and mood [6]. Therefore, an objective and straightforward test or scoring system may be more beneficial. Some objective tests are a 6 min walking test, walking velocity, and psoas muscle measurements [12].

The European Association for Cardio-Thoracic Surgery (EACTS) and the European Association of Preventive Cardiology (EAPC) of the European Society of Cardiology (ESC) have recently published pre-interventional frailty assessments for patients scheduled for cardiac surgery [13]. Whilst there are many frailty assessment tools that can be used, some of the most commonly described ones are gait speed, the Clinical Frailty Scale (CFS), Fried Frailty Criteria, deficit accumulation frailty index (FI), and Short Physical Performance Battery (SPPB). (Table 1).

1. Gait speed

Gait speed can be assessed in two parts: (1) the time required to walk a given distance of 4 or 5 m at a steady walking speed and (2) an endurance test assessing the distance covered within a 6 min walk test. Gait speed is an easily accessible assessment tool that can be assessed on its own or as part of a multicomponent frailty assessment (Green Score or Comprehensive Assessment of Frailty test). Multiple studies have identified gait speed as being a predictor of operative mortality in cardiac surgery [14]. More specifically, slow walkers, defined as having a gait speed of less than 0.83 m per second, were found to be a predictive risk factor for short-term (<30 days), intermediate (between 30 days and 1 year), and long-term (>1 year) mortality [14,15,16,17,18,19,20,21,22,23].

2. Clinical Frailty Scale

The Clinical Frailty Scale (CFS) is a global assessment tool that evaluates the level of fitness and frailty based on specific domains. Kenneth Rockwood developed the seven-point CFS in 2005 as part of the Canadian Study of Health and Aging (CSHA). It was later modified in 2007 to a nine-point scale [24,25]. These domains are used to generate a score from 1 (very fit) to 9 (terminally ill) and comprise the patient’s functional ability, cognitive status, comorbidities, and physical fitness [26]. Multiple studies have classified a CFS score of ≥5 as being frail [26,27]. Several studies have reported that higher CFS scores are associated with an increased risk of hospital mortality in patients undergoing cardiac surgery [13,27,28].

3. Fried frailty phenotype

This geriatric frailty phenotype evaluates frailty using five characteristics: poor endurance, slow gait, unintentional weight loss, weak grip strength, and low physical activity. Based on the total score, individuals are categorised into one of three frailty stages: no frailty (score of 0), pre-frailty (score of 1–2), and frailty (score of 3–5) [27,29]. A recent meta-analysis assessed post-operative outcomes using the Fried or modified Fried frailty indexes in open cardiac surgical procedures and demonstrated that frail patients had double the risk of all-cause mortality at one year post cardiac surgery [30].

4. Deficit accumulation frailty index

Kenneth Rockwood also developed the frailty index, based on the idea that frailty arises from the accumulation of many small deficits, rather than a single source [31]. As individuals age, they accumulate health deficits. Unsurprisingly, a larger number of deficits correlates with an increased risk [32,33]. This can be quantified through the frailty index, which calculates the total burden of health deficits across various clinical domains: some of which include diagnoses, cognitive function, disability, and physical function [34]. Frailty can be categorised as mild to moderate (FI 0.25–0.40) and severely frail (FI > 0.40) [34].

5. Short Physical Performance Battery (SPPB)

The SPPB is a three-part physical performance-based test that evaluates three timed tasks: (1) standing balance, (2) repeated chair stands, and (3) gait speed [35]. It is an objective tool to assess lower extremity physical function. Multiple studies have suggested using a SPPB score of ≤8 in males and ≤7 in females as a measure to identify functional decline by a deterioration in physical performance [36,37].

In addition to this, frailty has been linked to the accumulation of advanced glycation end-products (AGEs), a heterogeneous group of compounds that build up in various tissues as a physiological response to ageing and are potential biomarkers of biological skin [38]. More specifically, the accumulation of higher skin AGEs has been reported to be associated with frailty, as indicated by higher scores on Fried’s criteria and an elevated frailty index [38]. As Skin Auto Fluorescence (SAF) can assess AGEs non-invasively, it has been reported that elevated SAF levels are linked to frailty in older cardiac surgery patients and are associated with an increased risk of mortality or disability. This biomarker has the potential to enhance pre-operative risk assessment in cardiac surgery [39]. 

With the growing focus on patient-centred care, the continued advancement of frailty assessments holds the potential to enhance the quality of the information provided to patients by their surgeons and anaesthetists prior to their procedures, thereby strengthening the informed consent process [40]. 

**Table 1:** Frailty measurement tools.

	Frailty Measurement Tools	Description	Components	Frailty Defined as
1	Clinical Frailty Score (Rockwood) [24,25,26,27]	Evaluates frailty and overall fitness using clinical judgement, assessing the patient based on mobility, independence, and physical activity.	(1) Very fit.	Score of ≥5
			(2) Well: no active disease symptoms, exercise/active ocassionally.	
			(3) Manageing well: medical problems well controlled, not regularly active.	
			(4) Vulnerable: not dependent on others, often symptoms limit activities.	
			(5) Mildly frail: more evident slowing, need help in high-order instrumental ADLs.	
			(6) Moderately frail: requires help with all outside activities and keeping house. Inside, they often need help with bathing.	
			(7) Completely dependent for personal care.	
			(8) Very severely frail: completely dependent, approaching the end of life.	
			(9) Terminally ill: approaching the end of life. Life expectancy <6 months.	
2	Fried frailty phenotype [27,29]	Evaluates physical frailty.	Assess frailty using five characteristics: poor endurance, slow gait, unintentional weight loss, weak grip strength, and low physical activity.	Score of 1–2 = pre-frailty
				Score of 3–5 = frailty
3	Deficit accumulation frailty index (Rockwood) [31,32,34]	Quantifies frailty based on the accumulation of health deficits.	Quantifies the total burden of health deficits across multiple clinical domains (e.g., diagnoses, disability, cognitive function, and physical function) as a proportion.	Pre-frail (FI 0.15 ≤ 0.25)
				Mild to moderate (FI 0.25–0.40)
				Severely frail (FI > 0.40)
4	Short Physical Performance Battery [36,37]	Three-part physical performance-based test that evaluates three timed tasks.	(1) Standing balance.	Score of ≤8 (male) and ≤7 (female)
			(2) Repeated chair stands.	
			(3) Gait speed.	
5	Gait speed [16]	Assessed on its own or as part of a multicomponent frailty assessment.	(1) The time required to walk a given distance of 4 or 5 m at a steady walking speed.	Gait speed of <0.83 m per second
			(2) An endurance test assessing the distance covered within a 6 min walk test.	
6	Comprehensive assessment of frailty (CAF) score [10]	Includes age-related factors in addition to clinical and laboratory data to assess the perioperative risk.	Combination of characteristics of (1) Fried criteria: weakness, self-reported exhaustion, slowness of gait speed, and activity level; (2) physical performance: standing balance and body control; and (2) laboratory results: serum albumin, creatinine, brain natriuretic peptide (BNP), and forced expiratory volume in 1 s (FEV1).	Moderately frail: 11–25 points
				Severely frail: 26–35 points

7	Johns Hopkins Adjusted Clinical Groups frailty indicator [41]	Instrument based on 10 clusters of frailty-defining diagnoses.	Malnutrition, dementia, impaired vision, decubitus ulcer, incontinence of urine, loss of weight, poverty, barriers to access to care, difficulty in walking, and falls.	Presence of ≥1 diagnostic clusters
8	Frailty predicts death one year after cardiac surgery test (FORECAST) score [42]	A simplified version of the CAF using 5 components.	Chair rise × 3.	Score > 5
			Subjective reported weakness.	
			Serum creatinine.	
			Stair climb assessment.	
			Clinical Frailty Scale (scored by two doctors).	
9	Essential Frailty Toolset (EFT) [43]	A combination of physical, cognitive, and biochemical markers.	Timed chair rises (lower-extremity muscle weakness).	0 (least frail) to 5 (most frail)
			Mini-mental status examination (cognitive impairement).	
			Serum albumin (hypoalbuminaemia) and haemoglobin (anaemia).	

## 5. Impact on Surgical Outcomes

Frailty does not only influence short-term outcomes, but it also has a lasting impact on long-term morbidity and mortality.

### 5.1. Primary Outcomes: Short-Term and Long-Term Mortality

Many studies have reported the association between frailty and mortality risk [2,12,14,15,28,40,41,44,45,46,47]. A recent binational cohort study assessing 46,928 patients who underwent cardiac surgery in Australia and New Zealand reported a correlation between increased Clinical Frailty Scale score and increased hospital mortality [28]. Similarly, a meta-analysis carried out by Lee et al. (2021) [12] described the association between frailty and pre-frailty, using a combination of the Fried/modified Fried indices, deficit index, Bespoke Frailty Score, Clinical Frailty Score, Katz Index, walking velocity, and 6 min walk test, in patients undergoing cardiac surgery. After adjusting for baseline differences, frail patients were reported to be at double the risk of operative mortality compared to non-frail patients. Comparably, pre-frail patients carried a 1.5× increased risk of operative mortality relative to non-frail patients. However, they report no difference in long-term mortality between the risk-adjusted patient cohorts [12]. An updated meta-analysis (2023) [2] confirmed increased short-term mortality (<30 days) in frail individuals who underwent coronary artery or valvular surgeries/procedures when compared to robust patients. Additionally, frailty was associated with nearly a four-fold increased risk of midterm mortality, at 6 months to 1 year follow-up, relative to non-frail patients. In another study by Tran et al. (2018) [41] assessing the long-term follow-up in 40,083 patients undergoing CABG surgery, 22% were frail patients who were defined using the Johns Hopkins Adjusted Clinical Groups frailty indicator, a multidimensional instrument validated for research using administrative data. At 4 ± 2 years of follow-up, frailty was independently associated with a greater risk of long-term mortality. More specifically, there was a higher disparity in patient survival in patients less than 74 years old compared with patients greater than 85 years of age. Another study using the comprehensive assessment of frailty (CAF) score and the frailty predicts death one year after cardiac surgery test (FORECAST) score reported the association between higher frailty scores and short- and mid-term (up to 1 year) mortality post elective cardiac surgery, which was independent of age. As frailty scores were weakly associated with age, unlike the STS and EuroSCORE II, they could be used to highlight patients’ biological age [45]. 

### 5.2. Length of Stay in Hospital

Multiple studies have reported a positive correlation between frailty (using different measurement tools including a Frailty Index score of ≥3, Clinical Frailty Scale score of ≥5, and Edmonton Frail Scale) and a longer duration of stays in the intensive care unit (ICU) after CABG and/or valve surgery [15,28,40,44,47,48]. Similarly, several reports described the association between frailty (using various frailty scores: the Essential Frailty Toolset (EFT), comprehensive assessment of frailty (CAF) score, and Adjusted Clinical Groups frailty indicator) and a prolonged (≥14 days) length of hospital stays in patients post proximal aortic aneurysm surgery, CABG, and/or valve surgeries [14,46,49].

### 5.3. Secondary Outcomes

The post-operative complications in frail patients vary in the literature. A meta-analysis of 66,448 patients by Lee et al. (2021) [12] reported an increased risk of perioperative stroke and sternal wound complications amidst frail patients undergoing cardiac surgery. In this meta-analysis, frailty was defined using a combination of various frailty scores including the Fried/modified Fried indices (25%), deficit index (17%), Bespoke Frailty Score (17%), Clinical Frailty Score (8%), and Katz Index (8%). Other studies have reported an increased incidence of major adverse cardiac events, stroke, and major and acute kidney injury in frail patients post-cardiac surgery [49]. A recent binational cohort study of 46,928 patients (Ahuja 2024) [28] described the association between increasing Clinical Frailty Scale Score and the need for renal replacement therapy, tracheostomy, and increased duration of mechanical ventilation. Contrastingly, an observational cohort study by Ad et al. (2016) assessing 167 patients described no differences in the incidence of permanent stroke, atrial fibrillation, or renal failure between non-frail and frail patients, defined by the Cardiovascular Health Study Frailty Index criteria [15]. This may be attributable to the limited sample size of the patient cohort.

A recent meta-analysis in 2023 analysed the data of nineteen studies that reported post-operative complications in frail patients undergoing heart surgery and described a greater incidence of post-operative delirium, prolonged ventilation of >24–48 h, acute kidney injury, major bleeding, and reoperation due to bleeding. However, there was no significant correlation between frailty and the incidence of vascular complications or atrial fibrillation [2]. 

Similarly, an observational study reported a three- to eight-fold increased risk of post-operative delirium in frail patients who underwent elective cardiac surgery, depending on the definition of frailty that was utilised. Frailty was defined using the SPBB, 35-item frailty index (FI), and modified Fried criteria (MFC) score in this patient cohort [50]. They described weak hand grip strength and weight loss as specific features of frailty that were most associated with delirium [50]. 

### 5.4. Discharge Need for Rehabilitation

Frailty has an impact on the prognosis and rehabilitation course following cardiac surgery. Patients who are classified as frail have been found to have higher rates of discharge into an intermediate care facility post cardiac surgery, e.g., to a skilled nursing facility or temporary hospital, in comparison to robust patients [2,12,49]. A recent meta-analysis by Wong et al. (2023) [2] evaluated eight studies that reported data on discharge disposition and hospital readmission. Whist pre-frail patients were defined slightly differently across the studies, they were generally described as being in ‘an intermediate state between robustness and frailty’. Pre-frailty was not associated with hospital readmission following discharge from cardiac surgery; however, frailty was linked with increased hospital re-admission in patients post cardiac surgery [2]. Due to the use of several different frailty measurement tools, significant heterogeneity was observed across the studies and a random-effects model was used to calculate the summary effect estimates [2]. 

### 5.5. Quality of Life

Lytwyn et al. (2017) [51] conducted a study assessing functional survival, which was described as being alive at 1-year post cardiac surgery with a health-related quality of life score of greater than 60 on the EuroQol-Visual Analogue Scale. They recognised a correlation amid pre-operative frailty (defined as ≥3 of the modified Fried criteria, a Short Physical Performance Battery Score of ≤9, and a Clinical Frailty Scale of ≥3) which was found to be associated with a 2- to 3.5-fold increased risk of poor functional survival at 1-year post cardiac surgery. Therefore, their research team proposed an addition of frailty to EuroSCORE II to identify patients at risk of poor functional survival at 1-year following cardiac surgery [51]. 

## 6. Optimisation of Frail Patients Undergoing Cardiac Surgery

### 6.1. Pre-Operative Interventions

As there is an increase in the number of older and frail patients undergoing heart operations, it is important to carry out a frailty assessment early on and optimise the patient in preparation for their surgery [3]. Risk factors for post-operative complications can be modified if they are identified early, and the relevant pre-operative interventions are undertaken prior to surgery [52] (Table 2). 

#### 6.1.1. Multidisciplinary Geriatric Co-Management

The POPS (Perioperative Medicine for Older People Undergoing Surgery) service is established in certain units to improve surgical outcomes for older patients [53,54]. It follows a Comprehensive Geriatric Assessment (CGA) method, with a geriatrician-led multidisciplinary team to provide pre-operative optimisation, post-operative care, and rehabilitation planning [53]. The multidisciplinary team consists of geriatric physicians, nurses, anaesthetists, surgeons, physiotherapists, occupational therapists, and nutritionists [55]. The CGA is an evidence-based method that develops an individualised care plan with the aim to improve physical, functional, and social matters [56]. By utilising pre-operative assessments, perioperative care is optimised by implementing targeted interventions, some of which include medication reviews, nutritional support, and specialist geriatric consultations [47]. A recent meta-analysis reported that the CGA reduced the rate of post-operative delirium in individuals aged 65 and older undergoing surgery [47]. More specifically, Paille et al. (2021) [56] assessed the association between pre-operative CGA and the length of hospital stays post cardiac surgery. After propensity-score matching, pre-operative CGA was associated with a reduced length of stays in the hospital and in the intensive care unit. The reported effectiveness of CGA on other post-operative outcomes may be limited due to the fact that more patients who undergo a comprehensive assessment of perioperative risk may select conservative treatment [47]. 

Similarly, other units incorporate the ‘Heart Team’, which is a multidisciplinary group of cardiologists, cardiac surgeons, anaesthetists, ICU physicians, and specialists such as geriatricians who play an essential role in the multidisciplinary care of frail patients undergoing cardiac surgery [11]. A recent systematic review reported low-quality evidence which suggests that geriatric care may be associated with lower complication rates and an improved quality of life in older cardiac patients [57,58]. Addressing frailty and geriatric issues will ensure that the care provided is aligned with the patient’s wishes through a shared decision-making approach as this can influence the patient’s perceived quality of life, surgical outcome, and better patient-oriented outcomes [11]. 

#### 6.1.2. Nutrition

When pre-operative screening identifies the underlying factors contributing to frailty, the anaesthetist and surgical team can target optimising the specific areas pre-operatively. If nutrition is a concern, the patient can be advised to make lifestyle changes to optimise their nutritional status [3,59]. A recent single-centre study investigated the relationship between comprehensive geriatric assessment (including functional frailty, malnutrition, and anaemia) and post-operative recovery, measured by days alive and out of the hospital at 30 days (DAOH30), in 437 older patients undergoing cardiac surgery. The results showed that older age, cognitive dysfunction, emotional dysregulation, physical decline, malnutrition, and anaemia were associated with shorter DAOH30, with malnutrition having the strongest adverse impact. These findings highlight the importance of addressing targeted nutritional interventions to improve outcomes in this patient population [60]. 

#### 6.1.3. Cardiac Prehabilitation

With increasing patient complexity and co-morbidity in the cardiac surgery patient cohort, innovative strategies are needed to reduce the risk of adverse outcomes [3]. Cardiac prehabilitation implements the strategies used in cardiac rehabilitation proactively, instead of reactively [52,61]. It is a set of interventions carried out to improve patients’ nutritional status, physical capacity, and/or mental status to “defrail” elective patients [62]. There is noteworthy evidence that in various areas (aerobic conditioning, diabetic control, respiratory muscle training, lifestyle modification, psychoeducation, and sleep), intervening prior to cardiac surgery can improve outcomes [52]. For example, previous studies have reported the association of aerobic conditioning programmes and post-operative non-invasive ventilation requirements with a reduced length of stay in the ICU and hospital [52,61,63]. At present, the PREHAB study is an ongoing multicentre randomised controlled trial, “Pre-operative Rehabilitation for Reduction of Hospitalization After Coronary Bypass and Valvular Surgery”, aiming to examine the effect of prehabilitation in which frail patients (defined as patients aged 60 years or older with a CFS ≥ 3 and ≤7 at the time of acceptance for cardiac surgery) awaiting cardiac surgery either receive the current standard of care or participate in an 8-week exercise/education programme at a community-based cardiac rehabilitation centre [62]. 

#### 6.1.4. Decision for Non-Operative Management

The incorporation of a pre-operative multidisciplinary team (MDT) plays a key role in influencing the patient’s perioperative care plan. This is particularly important when considering the timing and type of surgical intervention, including the option not to undergo surgery and select lesser invasive percutaneous procedures and/or medical management for coronary or valvular pathology [11,12,64]. A recent study reported that pre-operative MDT care for frail patients undergoing cardiac surgery was associated with adjustments in surgical management to reduce the risk of severe complications, whereby 21% of patients underwent minimally invasive procedures and 10% received conservative treatment instead of open surgery [65]. Distinguishing between patients with advanced cardiovascular disease who may benefit from interventions and those with end-stage disease where such treatments offer little value is essential [66,67]. Shared decision making is “a process that involves the patient and the provider making collaborative decisions on the treatment plan, accounting for both clinical evidence and patient preferences” [67]. It enhances decision quality by adopting a patient-centred approach that actively involves and empowers patients as equal counterparts in their care [67]. Clinicians must carefully balance the principles of beneficence (acting in the patient’s best interest) with non-maleficence (avoiding unnecessary harm), while upholding the fundamental ethical duty to respect patient autonomy [68]. Given that cardiac surgery is an invasive surgical procedure, frail patients may benefit more from lesser invasive percutaneous procedures for coronary or valvular disease [12], such as percutaneous coronary intervention (PCI), transcatheter aortic valve replacement (TAVI), and transcatheter edge-to-edge mitral valve repair [69,70,71]. Patients whose goals prioritise comfort and functional status may select less invasive procedures or optimal medical therapy if an intervention’s risks outweigh its potential benefit.

### 6.2. Post-Operative Interventions

As frail patients are at increased risk of post-operative complications (some of which include increased pain, nutritional deficits, delirium, major adverse cardiac/cerebrovascular events, and pulmonary complications), it is important to develop realistic expectations with the frail patient and their family to support their recovery and reintegration into their daily activities, particularly regarding the potential for functional decline [3,40]. There are several interventions that can be incorporated to enhance the post-operative care of frail patients.

#### 6.2.1. Nutrition

Frail patients often have reduced muscle mass, making it important to collaborate with a dietitian to address their nutritional needs. Introducing a balanced diet with appropriate nutritional supplementation early in the post-operative period along with establishing a comprehensive long-term nutrition plan is important for the patient’s recovery and overall well-being [3,59]. These patients may also benefit from protein and iron supplementation after surgery [3]. 

#### 6.2.2. Post-Operative Delirium

Another important intervention is the early recognition, prevention, and treatment of post-operative delirium. All post-operative patients should be screened for post-operative delirium during the first three days post-operatively and until their clinical condition stabilises, using validated tools such as the 4AT and CAM-ICU scales [11]. The American College of Surgeons and the American Geriatrics Society recommend minimising deliriogenic medications (amitriptyline and benzodiazepines) and addressing pain management using a multimodal approach, with an emphasis on opioid-sparing techniques for pain management where possible [3,72]. Multi-modal non-pharmacological methods, such as the Hospital Elderly Life Program (HELP) bundle, have been reported to be an effective strategy to reduce delirium in general surgery patients. This programme focuses on increased mobility, sleep enhancement, orientation protocols, hearing and vision optimisation, and avoidance of dehydration [60,73,74]. Whilst it has not been investigated in frail patients undergoing cardiac surgery, by inference, it may be beneficial in this patient cohort.

#### 6.2.3. Rehabilitation

Early planning for cardiac rehabilitation should be implemented, incorporating early patient ambulation, mobility support, fall precautions, and frequent repositioning to avoid pressure sores [3,75]. A recent study reported that the CFS assessment could be utilised in the cardiac rehabilitation setting, as it can be performed in less than one minute and has a significant correlation with assessments of functional capacity, activities of daily living, and clinical parameters [76]. Early rehabilitation, both in-hospital and in the immediate post-discharge period, aims to reduce the adverse effects of prolonged bed rest [77]. Moreover, it has been reported to decrease the risk of post-operative complications, enhance autonomic cardiac function at discharge, and shorten the length of hospital stay [78,79,80].

#### 6.2.4. Occupational Therapy

Early involvement of occupational therapists is important for ensuring timely, appropriate, and safe discharges of frail patients. Occupational therapists play an important role in promoting independent living as they focus on the patient’s day-to-day activities and support the patient’s engagement in daily tasks, which ultimately enhances the patient’s overall quality of life [81]. 

## 7. Conclusions

Frailty has become increasingly prevalent in a large proportion of patients undergoing cardiac surgery. It has been found to be associated with increased post-operative morbidity and mortality. Early identification of frail patients during the perioperative period is important for risk stratification. This would assist in identifying patients who would benefit from pre-operative and post-operative optimisation strategies. Incorporating frailty into risk assessment frameworks may enhance decision making with the goal of improving surgical outcomes, enhancing patient quality of life, and minimising avoidable harm. Further studies are required to confirm the best way of diagnosing frailty and to identify the most effective interventions to minimise the impact of frailty on outcomes following cardiac surgery.

## Figures and Tables

**Table 2:** Interventions to optimise frail patients undergoing cardiac surgery.

Pre-Operative	Post-Operative
Frailty Screening	Early recognition, prevention, and treatment of post-operative delirium
Multidisciplinary ‘Heart’ Team	Optimising nutrition
Geriatric Assessment	Early Cardiac Rehabilitation
Cardiac Prehabilitation	Early Occupational Therapy
Nutrition Status	

## Data Availability

No new data are presented in this narrative review.
